# Who was affected by the shortage of penicillin for syphilis in Rio de Janeiro, 2013–2017?

**DOI:** 10.11606/s1518-8787.2020054002196

**Published:** 2020-10-28

**Authors:** Rachel Sarmeiro Araujo, Ana Sara Semeão de Souza, José Ueleres Braga

**Affiliations:** I Universidade do Estado do Rio de Janeiro Instituto de Medicina Social Programa de Pós-graduação em Saúde Coletiva Rio de JaneiroRJ Brasil Universidade do Estado do Rio de Janeiro . Instituto de Medicina Social . Programa de Pós-graduação em Saúde Coletiva . Rio de Janeiro , RJ , Brasil; II Universidade do Estado do Rio de Janeiro Instituto de Medicina Social Departamento de Epidemiologia Rio de JaneiroRJ Brasil Universidade do Estado do Rio de Janeiro . Instituto de Medicina Social . Departamento de Epidemiologia . Rio de Janeiro , RJ , Brasil; III Fundação Oswaldo Cruz Escola Nacional de Saúde Pública Sergio Arouca Departamento de Epidemiologia Rio de JaneiroRJ Brasil Fundação Oswaldo Cruz . Escola Nacional de Saúde Pública Sergio Arouca . Departamento de Epidemiologia . Rio de Janeiro , RJ , Brasil

**Keywords:** Penicillin G Benzathine, provision & distribution, Spatiotemporal Analysis, Syphilis, drug therapy, Health Status Disparities, Ecological studies

## Abstract

**OBJECTIVE:**

To analyze the shortage of benzathine penicillin G (BPG), characterizing its temporal evolution and spatial distribution in the city of Rio de Janeiro from 2013 to 2017.

**METHODS:**

This ecological study used gestational and congenital syphilis notifications, BPG distribution records, and sociodemographic data from the population of Rio de Janeiro. To quantify the shortage, a BPG supply indicator was estimated per quarter for each neighborhood between 2013 and 2017. Thematic maps were created to identify areas and periods with greater BPG shortage, described according to sociodemographic factors, health services network, and epidemiological features in the incidence of syphilis.

**RESULTS:**

BPG shortage in Rio de Janeiro from 2013 to 2017 was not homogeneous in space nor in time. The temporal evolution and spatial distribution of BPG scarcity shows that the shortage affected the inhabitants of the municipality in different ways. Shortage was lower in 2013 and 2016 and more severe in 2014, 2015, and 2017, particularly in neighborhoods within the programmatic areas PA3 and PA5, poorer and with higher prevalence rates of gestational and congenital syphilis.

**CONCLUSIONS:**

Analyzing BPG shortage and its temporal evolution and spatial distribution in Rio de Janeiro allowed us to realize that the inhabitants are affected in different ways. Understanding this process contributes to the planning of actions to face shortage crises, minimizing possible impacts on the management of syphilis and reducing inequality in access to treatment.

## INTRODUCTION

Between 2014 and 2017 Brazil experienced the shortage of benzathine penicillin G (BPG), procaine and crystalline formulations, the main antibiotics for the treatment of syphilis that integrate the Basic Component of Pharmaceutical Assistance (BCPA) in the Unified Health System (SUS). The drug was scarce in pharmaceutical retail market, and the few available stocks in SUS were primarily intended to syphilis treatment, especially for gestational and congenital syphilis (CS) ^1^ .

Syphilis is a systemic, curable, and human sexually transmitted infection (STI) caused by the *Treponema pallidum* . Untreated syphilis in pregnancy results in a considerable amount of fetal and neonatal death and in a high likelihood of vertical transmission, especially during the primary and secondary stages of the disease ^2 , 3^ .

Rates of syphilis in pregnant women have increased in Brazil: in 2010, there were 3.5 gestational syphilis (GS) and 2.4 CS cases per 1,000 live births; in 2016, GS detection rate was 12.4 and CS incidence 6.8 per 1,000 live births ^4^ .

The 2016 Pan American Health Organization (PAHO) report informs that the increased CS incidence may be justified by the improvement in epidemiological surveillance, the broadening in the distribution of rapid diagnostic tests, the lack of penicillin, and patient referral to other care levels by basic health units instead of treating them, generating patient loss during the process ^5^ .

According to Nurse-Findlay et al. ^6^ , 39 countries reported shortage of BPG between 2014 and 2016, five of which belonged to Latin America (Brazil, Jamaica, Panama, Suriname, and Trinidad and Tobago). In Brazil, as in other countries, BPG scarcity stemmed from the combination of two factors: reduction of finished dosage forms (FDF) and the termination of quality certification for active pharmaceutical ingredient (API) ^6 , 7^ .

Despite the accurate diagnosis and effective low-cost treatment, CS is still a serious public health issue associated with many perinatal complications. CS is the second leading infectious cause of stillbirth globally and a preventable cause of infant morbidity and mortality that occurs primarily in low- and middle-income countries ^8^ .

As medicines shortages compromise the timely and adequate treatment of preventable diseases such as syphilis, understanding the distribution and characteristics of this phenomenon may minimize possible consequences for the population’s health. Considering that, this article analyzes the shortage of BPG, characterizing its temporal evolution and spatial distribution in the city of Rio de Janeiro from 2013 to 2017.

## METHODS

This mixed ecological study (multiple groups and multiple time periods) analyzed neighborhoods/regions and the quarters of the addressed years. Our study population was composed of cases of gestational syphilis (GS) reported by the *Sistema de Informação de Agravos de Notificação* (SINAN – Notifiable Diseases Information System) of the municipality of Rio de Janeiro, and benzathine penicillin G (BPG) distribution for these cases by place of residence (neighborhood) between 2013 and 2017. Only municipal health units (HU) participated in the study. That is, records from federal, state, university, private, and psychiatric hospitals from other administrative spheres were excluded.

**Figure 1:**
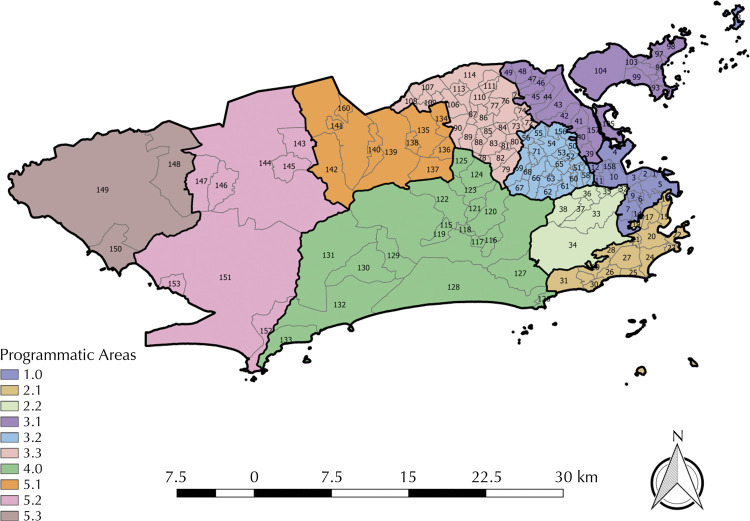
Map of the division of the municipality of Rio de Janeiro by Programmatic Areas of Health.

Note: Neighborhood codes: 1 - Saúde; 2 - Gamboa; 3 - Santo Cristo; 4 - Caju; 5 - Centro; 6 - Catumbi; 7 - Rio Comprido; 8 - Cidade Nova; 9 - Estácio; 10 - Imperial de São Cristóvão; 11 - Mangueira; 12 - Benfica; 13 - Paquetá; 14 - Santa Teresa; 15 - Flamengo; 16 - Glória; 17 - Laranjeiras; 18 - Catete; 19 - Cosme Velho; 20 - Botafogo; 21 - Humaitá; 22 - Urca; 23 - Leme; 24 - Copacabana; 25 - Ipanema; 26 - Leblon; 27 - Lagoa; 28 - Jardim Botânico; 29 - Gávea; 30 - Vidigal; 31 - São Conrado; 32 - Praça da Bandeira; 33 - Tijuca; 34 - Alto da Boa Vista; 35 - Maracanã; 36 - Vila Isabel; 37 - Andaraí; 38 - Grajaú; 39 - Manguinhos; 40 - Bonsucesso; 41 - Ramos; 42 - Olaria; 43 - Penha; 44 - Penha Circular; 45 - Braz de Pina; 46 - Cordovil; 47 - Parada de Lucas; 48 - Vigário Geral; 49 - Jardim América; 50 - Higienópolis; 51 - Jacaré; 52 - Maria da Graça; 53 - Del Castilho; 54 - Inhaúma; 55 - Engenho da Rainha; 56 - Tomás Coelho; 57 - São Francisco Xavier; 58 - Rocha; 59 - Riachuelo; 60 - Sampaio; 61 - Engenho Novo; 62 - Lins de Vasconcelos; 63 - Meier; 64 - Todos os Santos; 65 - Cachambi; 66 - Engenho de Dentro; 67 - Água Santa; 68 - Encantado; 69 - Piedade; 70 - Abolição; 71 - Pilares; 72 - Vila Kosmos; 73 - Vicente de Carvalho; 74 - Vila da Penha; 75 - Vista Alegre; 76 - Irajá; 77 - Colégio; 78 - Campinho; 79 - Quintino Bocaiuva; 80 - Cavalcanti; 81 - Engenheiro Leal; 82 - Cascadura; 83 - Madureira; 84 - Vaz Lobo; 85 - Turiaçu; 86 - Rocha Miranda; 87 - Honório Gurgel; 88 - Oswaldo Cruz; 89 - Bento Ribeiro; 90 - Marechal Hermes; 91 - Ribeira; 92 - Zumbi; 93 - Cacuia; 94 - Pitangueiras; 95 - Praia da Bandeira; 96 - Cocotá; 97 - Bancários; 98 - Freguesia (Ilha); 99 - Jardim Guanabara; 100 - Jardim Carioca; 101 - Tauá; 102 - Moneró; 103 - Portuguesa; 104 - Galeão; 105 - Cidade Universitária; 106 - Guadalupe; 107 - Anchieta; 108 - Parque Anchieta; 109 - Ricardo de Albuquerque; 110 - Coelho Neto; 111 - Acari; 112 - Barros Filho; 113 - Costa Barros; 114 - Pavuna; 115 - Jacarepaguá; 116 - Anil; 117 - Gardênia Azul; 118 - Cidade de Deus; 119 - Curicica; 120 - Freguesia (Jacarepaguá); 121 - Pechincha; 122 - Taquara; 123 - Tanque; 124 - Praça Seca; 125 - Vila Valqueire; 126 - Joá; 127 - Itanhangá; 128 - Barra da Tijuca; 129 - Camorim; 130 - Vargem Pequena; 131 - Vargem Grande; 132 - Recreio dos Bandeirantes; 133 - Grumari; 134 - Deodoro; 135 - Vila Militar; 136 - Campo dos Afonsos; 137 - Jardim Sulacap; 138 - Magalhães Bastos; 139 - Realengo; 140 - Padre Miguel; 141 - Bangu; 142 - Senador Camará; 143 - Santíssimo; 144 - Campo Grande; 145 - Senador Vasconcelos; 146 - Inhoaíba; 147 - Cosmos; 148 - Paciência; 149 - Santa Cruz; 150 - Sepetiba; 151 - Guaratiba; 152 - Barra de Guaratiba; 153 - Pedra de Guaratiba; 154 - Rocinha; 155 - Jacarezinho; 156 - Complexo do Alemão; 157 - Maré; 158 - Vasco da Gama; 159 - Parque Columbia; 160 - Gericinó; 161 - Lapa; 162 - Vila Kennedy.

The study comprised 160 neighborhoods and their respective programmatic areas (PA) of health ( Figure 1 ) registered in SINAN in the addressed period. Four data sources were used: (i) SINAN databases on SG and SC records for the municipality of Rio de Janeiro; (ii) BPG distribution reports and spreadsheets disclosed by the Pharmaceutical Assistance Center of the Municipal Department of Health (SMS-Rio); (iii) census tract-level and neighborhood-level population data from the 2010 Demographic Census of the Brazilian Institute of Geography and Statistics (IBGE); and (iv) data on live births records from the *Sistema de Informação de Nascidos Vivos* (SINASC – Live Birth Information System) of the municipality of Rio de Janeiro.

BPG supply for GS use was measured considering the ratio between two quantities: (a) the amount of BPG vials dispensed to each neighborhood health units; and (b) the necessary amount of BPG vials for treating pregnant women with syphilis and their respective partners in each neighborhood.

Data on BPG distribution for the HUs of each neighborhood of the municipality of Rio de Janeiro were collected from electronic spreadsheets, provided by the SMS-Rio, containing: (i) the amount of BPG vials dispensed to each neighborhood HUs; (ii) the amount of BPG vials dispensed to the General Coordination of the Programmatic Area Primary Care (CAP) and their respective covered neighborhoods; (iii) the amount of BPG vials specifically dispensed to each neighborhood HUs and for the CAP of the same coverage area.

The amount of vials dispensed to each neighborhood HUs was calculated based on the following estimates, according to data type: (i) for type 1 data, the amount of vials dispensed to each HU of a neighborhood was added together; (ii) for type 2, the amount of vials distributed to each HU was estimated based on the number of SG notifications; then the amount of vials in the HUs of each neighborhood was added together; (iii) for type 3, the two previous methods were combined.

As not all BPG provided to the HUs was destined to GS treatment, we assumed that 80% were used in the treatment of pregnant women with syphilis and their partners. Two reasons justify such premise: (i) BPG is used to treat other infections also prevalent in HUs, as rheumatic fever, upper respiratory tract infections, soft-tissue infections (erysipela, impetigo), and penicillin-susceptible *Streptococcus pneumoniae* pneumonias; (ii) BPG is used to contain the increase in CS cases, as SMS-Rio prioritized the use of BPG in the treatment of pregnant women with syphilis.

The dosage of each individual treatment was considered to determine the required amount of BPG vials to treat pregnant women and their respective partners. In the lack of data on disease clinical classification (staging), the 3-dose therapeutic regimen of BPG was regarded for the treatment of pregnant women – each using two vials (1,200,000 IU), totalizing six vials (7,200,000 IU).

The required amount of BPG vials to treat pregnant women was estimated from the total number of GS cases reported in the Hus of each neighborhood, multiplied by 6, and added to treated partners (who used three doses of BPG). After estimating the amount of dispensed and required vials, supply was calculated using the following formula:

$$Ab_{i}=\frac{X_{i}}{Y_{i}}$$

*Ab*_i_ – Neighborhood *i s* upply indicator;

*X*_i_ – Amount of BPG vials dispensed to treat GS and partners in neighborhood *i;*

*Y*_i_ *–* Amount of BPG vials required to treat pregnant women and partners in neighborhood *i.*

As we observed an uneven distribution throughout the year, BPG supply indicator was estimated per quarter. Our unit of time was the quarter of the year, as it represents BPG distribution in a more homogeneous way.

Supply indicator is a ratio whose values range from 0 to ∞. Thus, supply level was analyzed considering *Ab* values and other four important categories: (i) severe, when *Ab* value ranged from 0 to 0.49; (ii) substantial, if *Ab* ranged from 0.50 to 0.74; (iii) non-substantial, if *Ab* ranged from 0.75 to 0.99 and (iv) without shortage, when *Ab* was greater than or equal to 1.

Neighborhoods were characterized by grouping health services into four classes: (i) primary care services (family clinics, municipal health centers, and family health teams); (ii) secondary care services (polyclinics and psychosocial care center); (iii) emergency network (emergency units and regional emergency coordination); and (iv) hospitals.

Sociodemographic data on neighborhoods were also collected, such as: male-female ratio, proportion of population living in subnormal clusters, homicide rate, and Social Development Index (SDI). As for population health characteristics, live births rates, CS incidence, GS detection, and proportion of pregnant women with nontreponemal test were used.

The temporal evolution of BPG supply level in the municipality was analyzed using trend charts created in STATA 12.0. Thematic maps were created in QGIS 2.18.14 to describe the distribution by each quarter/year of the shortage of BPG in Rio de Janeiro neighborhoods.

Finally, neighborhoods were analyzed according to types of health service (total), sociodemographic status, and health conditions (mean, minimum, and maximum). Neighborhoods were grouped according to the PAs defined by SMS-Rio to characterize their areas. This research project was submitted and approved by the Research Ethics Committee of the SMS-Rio (CAAE: 06019018.3.3001.5279).

## RESULTS

**Figure 2:**
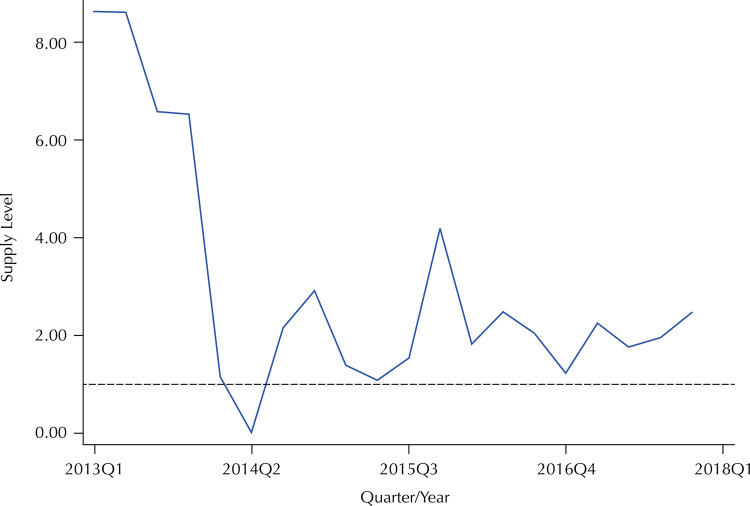
BPG supply level in the municipality of Rio de Janeiro second quarter-year, from 2013 to 2017.

Note: The dashed line indicates a supply ratio equal to 1.

Between 2013 and 2017, the municipality of Rio de Janeiro experienced considerable shortages of benzathine penicillin G (BPG). The years 2014 and 2015 had the lowest supply levels among other quarters of the study period, although 2017 also presented low levels ( Figure 2 ).

**Figure 3:**
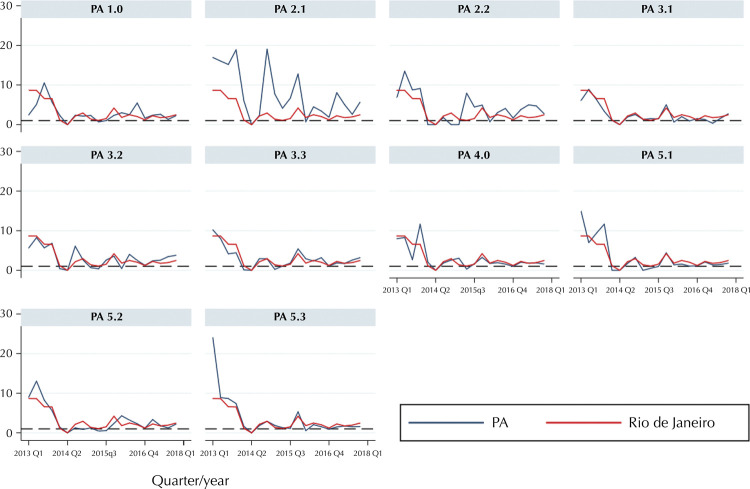
BPG supply level by programmatic area in the municipality of Rio de Janeiro second quarter-year, from 2013 to 2017.

Note: The dashed line indicates a supply ratio equal to 1.

At the beginning of the period, especially in 2013, BPG supply level in Rio de Janeiro neighborhoods was high. However, the following years experienced a decrease in most PAs neighborhoods. Shortages were lower in 2013 and 2016 and more severe in 2014, 2015, and 2017, particularly in PA3 and PA5 neighborhoods ( Figure 3 ).

PA2.1 and PA2.2 neighborhoods had supply levels above the municipal average in nearly every quarter of the study period, whereas other areas had levels below municipal average in 2014 to 2017. Supply levels were lower in 2014 and 2015, despite the severe shortages in PA3.1, PA3.2, and PA3.3 neighborhoods in 2015 and in PA5.1, PA5.2, and PA5.3 in 2017 ( Figure 3 ).

**Figure 4:**
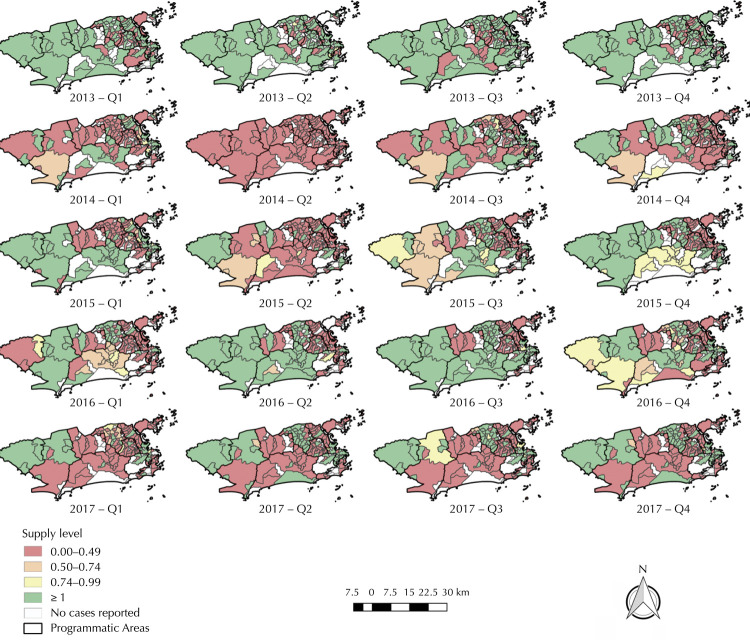
Benzathine penicillin G spatial distribution by neighborhoods in the municipality of Rio de Janeiro second quarter-year, from 2013 to 2017.

The spatial distribution of BPG supply level in Rio de Janeiro neighborhoods was heterogeneous for all years studied. However, the proportion of shortage in neighborhoods was lower in 2013 and higher in 2014. In 2013, shortage neighborhoods were mainly located in PA3.1, PA3.2, and PA3.3 ( Figure 4 ). In 2014, especially in the second quarter, we observed a near universal shortage, regardless of the higher concentration in PA3 and PA5 neighborhoods in other quarters and the lowest shortages levels in PA2.1 and PA2.2 neighborhoods.

The shortage observed in 2014 seems to extend into the second quarter of 2015 and decrease in 2016, especially in the two middle quarters of the year. However, similarly to other years studied, several PA3.1 and PA3.2 neighborhoods experienced shortages. This area expands and intensifies in 2017, reaching mostly PA5.2 and PA5.3 neighborhoods. In summary, the phenomenon distribution in all periods is mainly characterized by differences between southern (PA2), with little or no shortage, north (PA3), with high shortage, and west zones (PA5), with severe shortage neighborhoods, especially at the end of the study period ( Figure 4 ).

Regarding regional characteristics of the municipality of Rio de Janeiro, the number of HUs varied among regions, especially regarding primary care units, which are more concentrated in the north (PA3) and west (PA5) zones – although PA3 has more neighborhoods and is more populous and PA5 is larger and less inhabited ( Table ).

**Table:** Regional characteristics (programmatic areas) according to health service types and sociodemographic and health conditions of the municipality of Rio de Janeiro between 2013 and 2017.

Variables	AP 1.0	AP 2.1	AP 2.2	AP 3.1	AP 3.2	AP 3.3	AP 4.0	AP 5.1	AP 5.2	AP 5.3
Neighborhoods (N)	15	18	7	28	23	29	19	10	8	3
Health Services (N)										
Primary care services	16	11	9	26	20	26	12	20	31	23
Secondary care services	1	0	1	3	1	0	2	1	0	1
Emergency and urgent care networks	1	2	0	3	1	2	2	3	0	4
Hospital	5	2	3	2	3	4	4	1	0	0
Population characteristics*										
Sociodemographic										
Male-Female Ratio	0.92	0.81	0.81	0.89	0.87	0.87	0.91	1.18	0.95	0.93
	(0.82–1.06)	(0.74–0.97)	(0.77–0.91)	(0.83–0.98)	(0.78–1.31)	(0.79–1.06)	(0.84–0.98)	(0.88–3.81)	(0.90–1.02)	(0.92–0.93)
Proportion of population living in subnormal clusters	33.3	17.8	15.8	28.5	15.8	22.9	21.8	13.8	12.3	13.2
	(0–79.4)	(0–100)	(0–43.5)	(0–84.3)	(0–85.8)	(0–92.1)	(0–68.2)	(1.8–36.0)	(0–27.6)	(3.0–22.8)
Homicide rate	119.4	33.3	47.1	71.9	81.9	128.9	85.1	94	94.8	110.0
	(38.8–224.3)	(0–89.4)	(7.9–137.4)	(0–229.8)	(25.4–226.4)	(18.6–404.3)	(22.8–175.0)	(6.6–175.2)	(39.2–189.7)	(88.4–137.1)
Social Development Index	0.59	0.71	0.65	0.60	0.61	0.59	0.58	0.58	0.57	0.54
	(0.55–0.63)	(0.54–0.8)	(0.54–0.7)	(0.53–0.71)	(0.54–0.68)	(0.54–0.65)	(0.31–0.76)	(0.55–0.64)	(0.5–0.72)	(0.53–0.55)
Health										
Live birth rate	244.6	112.6	60.6	107.3	151.8	82.4	164.1	96.7	86.3	32.4
	(14.6–1.327.6)	(1.7–930.5)	(44.9–83.3)	(0.15–727.6)	(8.9–532.9)	(1.7–485.5)	(2.8–1.524.5)	(0.2–275.1)	(0.06–273.4)	(3.2–74.8)
Congenital syphilis incidence rate	23	13	13.8	428.9	16.3	17.8	15.7	18.7	2609.6	21.3
	(2.1–35)	(1–33.4)	(4.9–35.2)	(0.27–1.1494)	(2.2–54.2)	(2.9–44.3)	(0.76–42.8)	(4.2–30.4)	(4.6–1.8158)	(14.2–25)
Gestational syphilis detection rate	47.1	26.8	23.7	1.061.6	39.7	38.3	30.3	42.3	3.066.3	40.1
	(10.4–109.7)	(4–65.8)	(6.1–81.8)	(3–2.8735)	(6.8–169.8)	(4.5–89.7)	(2.2–70.3)	(4.2–76.5)	(10.2–24.211)	(29.6–47.2)
Proportion of pregnant women with nontreponemal test	0.58	0.58	0.82	0.70	0.66	0.65	0.38	0.64	0.41	0.47
	(0–0.83)	(0–1)	(0.62–1)	(0–1)	(0.33–0.90)	(0–0.91)	(0–1)	(0–0.85)	(0–0.65)	(0.40–0.59)

* Mean values (minimum – maximum).

South zone (PA2) neighborhoods have better SDI, less violence, fewer households in subnormal clusters, and lower male/female ratio. The north and west zones present the worst socioeconomic status ( Table ).

South zone neighborhoods significantly differ from other areas of the municipality regarding live birth rates and the proportion of pregnant women who underwent laboratory tests to diagnose syphilis during pregnancy. Such indicator reflects the quality of the surveillance system of HUs located in Rio de Janeiro neighborhoods ( Table ).

## DISCUSSION

The shortage of benzathine penicillin G (BPG) in Rio de Janeiro from 2013 to 2017 was not homogeneous in space nor in time. Whereas north zone neighborhoods experienced a severe shortage in 2014, neighborhoods in the south and the south-central region (which shares borders with the latter) barely suffered. The year of 2017 brought yet another shortage, this time incorporating the west zone as well.

Among the main factors that may have contributed to this scenario, Brazil and international countries experienced, especially in 2014, a shortage of BPG due to the lack of the raw material used in its manufacturing ^1 , 3 , 5^ . A small number of global manufacturers rely on the active pharmaceutical ingredient (API), representing one of the causes of the lack of penicillin in the world. At least five companies abandoned the global penicillin market in the last ten years in search for a more profitable drug ^5^ .

Today, just four companies produce the active pharmaceutical ingredient for penicillin – one Austrian and three Chinese. These companies have recently limited their production to 20% of their capacity because the drug become cheaper. Regardless of its strong pharmaceutical industry, Brazil stopped manufacturing API for several drugs in the early 1990s, including penicillin. The national slowdown in API manufacturing ensues in 90% of the sector demands to be currently met by imports ^11^ .

At the end of 2015, the Brazilian Health Regulatory Agency (ANVISA) withdrew the good practices certificate from the API supplier for penicillin manufacturing. It was only by end of 2016 that ANVISA granted a waiver, allowing two FDF laboratories to purchase the API. However, both FDF manufacturing significantly delayed production and re-entry into the market as they needed time to show equivalence and receive marketing authorization with the new supplier ^6 , 12^ .

Irregularity within API supply (the main raw material) and the supply of products outside acceptable quality standards seem to be the main reasons for medicines shortage, as they tend to interrupt production process ^13^ .

This issue enhances when manufacturers are reduced or limited to a single supplier. Brazil, which does not produce most drugs on industrial scale and depends on the international market, is put in a fragile situation ^13^ .

According to a report published by the Ministry of Health in July 2015, BPG was scarce in almost half of the Brazilian states. Its stock was depleted in 11 states (41%), mostly in north and northeast regions. Other areas, including Rio de Janeiro State, were also experiencing shortage ^14^ .

BPG manufacturing was expected to be regularized as of July 2014, which did not occur, so that the medicine shortage was still an issue in 2016 and 2017. The shortage of BPG reached 61% of Brazilian states. In the case of crystalline penicillin, it reached 100% in March 2016 ^1^ .

Similarly to the national scenario, our study reveals that the lowest BPG supply levels in the municipality of Rio de Janeiro occurred in 2014 and 2015. However, this phenomenon was heterogeneous among the municipality programmatic areas (PA), being more pronounced in PA3 (north) and PA5 (west).

PA3.1, PA3.2, and PA3.3 together cover half of the neighborhoods in the municipality of Rio de Janeiro, besides presenting higher average homicide and live birth rates compared with other areas. In turn, PA5.1, PA5.2, and PA5.3 have the largest territorial extension and, on average, the highest congenital (CS) and gestational syphilis (SG) rates and the lowest social development index (SDI) values.

Domingues et al. (2014) ^15^ conducted a study between 2011 and 2012 with 23,894 Brazilian women and found that pregnant women with up to seven years of education had 3.2 more chances of contracting syphilis than those with a higher education level. Similarly, Mosque et al. (2012) ^16^ evaluated the epidemiological profile of syphilis in pregnant women in the city of Sobral (CE) from 2006 to 2010 and found that the disease was more prevalent among pregnant women who only attended from 5th to 8th grade of elementary school.

Other studies reported a higher incidence of CS among social strata with lower education level, socioeconomically disadvantaged racial/ethnic groups (blacks), and with worse living conditions ^17 , 18^ . Such factors reinforce the occurrence of syphilis cases among pregnant women with low education level and in areas of worse socioeconomic conditions in crisis situations, such as BPG shortage, as observed in our study.

Another relevant factor on socioeconomic status and syphilis is its high vertical transmission rate. Domingues et al. (2014) ^19^ reported a 34.3% vertical transmission rate, in which the likelihood of a pregnant woman having a CS outcome was sixteen times higher if she had a lower education level. The intensity of syphilis vertical transmission differs according to stage maternal disease. However, the appropriate penicillin treatment can prevent 97% of vertical transmission cases ^20^ . Considering the difficulties in covering syphilis screening during pregnancy and the pregnant woman and her partner adherence to treatment, medicine shortage impacts CS control.

In 2016, 58.1% of pregnant women with syphilis received inadequate treatment in Brazil, 26.5% received no treatment, 11.3% of the cases were ignored, and only 4.1% were adequately treated ^3^ . Most pregnant women who received none or inadequate treatment may transmit the infection to their concepts, possibly leading to fetal and neonatal death, prematurity, low birth weight, or congenital infection ^21^ .

In 2016 Taylor et al. ^20^ estimated the required amount of BPG to treat pregnant women diagnosed with syphilis to face the CS epidemic in countries with high disease burden. Their results indicate that, if the World Health Organization recommendations for eliminating CS are adopted, these countries require a BPG amount twice as high as that predicted annually, reaching at least 95% coverage of syphilis screening.

The main limitations in our study are related to data availability on BPG distribution in health units, distribution record in the municipal health network, and the method adopted to estimate BPG supply level. The increased incidence of GS and CS in the studied period indicates that, although plausible, underreporting did not prevent us from detecting worsening in the epidemiological situation. It is also unlikely that major variations in time and space have occurred in the supposed underreporting.

## FINAL REMARKS

Analyzing BPG shortage and its temporal evolution and spatial distribution in Rio de Janeiro allowed us to realize that inhabitants are affected in different ways. Understanding this process contributes to the planning of actions to face shortage crises, minimizing possible impacts on the management of syphilis and reducing inequality in access to treatment.

Seeking long-term solutions to ensure the supply of penicillin in SUS is an evident and continuous need, as this would allow an adequate treatment to syphilis and avoid new shortage crises. Penicillin, in procaine and crystalline formulation, is essential to treat congenital syphilis and other infections, reducing neonatal mortality. This subject requires further studies, as the impact of BPG shortages on the significant increase in CS cases has been little explored.
