# Supplementary material for: Who was affected by the shortage of penicillin for syphilis in Rio de Janeiro, 2013–2017?
Source: Rev Saude Publica. 2020 Oct 28;54:109. doi: 10.11606/s1518-8787.2020054002196 (PMC7593023; doi:10.11606/s1518-8787.2020054002196)
Supplement: Translation

# Translation: A quem afetou o desabastecimento de penicilina para sífilis no Rio de Janeiro, 2013–2017?

**Authors:** Rachel Sarmeiro Araujo, Ana Sara Semeão de Souza, José Ueleres Braga

**Affiliations:** I Brasil Universidade do Estado do Rio de Janeiro. Instituto de Medicina Social. Programa de Pós-graduação em Saúde Coletiva. Rio de Janeiro, RJ, Brasil; II Brasil Universidade do Estado do Rio de Janeiro. Instituto de Medicina Social. Departamento de Epidemiologia. Rio de Janeiro, RJ, Brasil; III Brasil Fundação Oswaldo Cruz. Escola Nacional de Saúde Pública Sergio Arouca. Departamento de Epidemiologia. Rio de Janeiro, RJ, Brasil

**Keywords:** Penicilina G Benzatina, provisão & distribuição, Análise Espaço-Temporal, Sífilis, tratamento farmacológico, Disparidades nos Níveis de Saúde, Estudos Ecológicos

## Abstract

**OBJETIVO:**

Analisar o desabastecimento da penicilina benzatina (PB), caracterizando sua evolução temporal e distribuição espacial no município do Rio de Janeiro de 2013 a 2017.

**MÉTODOS:**

Trata-se de estudo ecológico misto realizado com notificações de sífilis gestacional e congênita, registros de distribuição de PB e de dados sociodemográficos da população dos bairros do município do Rio de Janeiro. Para mensurar o desabastecimento foi calculado por trimestre um indicador de abastecimento de PB para cada bairro, entre 2013 e 2017. Mapas temáticos foram produzidos para identificar áreas e períodos com maior desabastecimento de PB, o qual foi descrito segundo condições sociodemográficas, rede de serviços de saúde e aspectos epidemiológicos da incidência de sífilis por bairro.

**RESULTADOS:**

O desabastecimento de PB no município do Rio de Janeiro, no período de 2013 a 2017, não foi homogêneo no espaço ou no tempo. A evolução temporal e a distribuição espacial da escassez de PB revelam que o desabastecimento afetou de formas distintas os habitantes do município, sendo menor em 2013 e 2016 e mais intenso em 2014, 2015 e 2017, principalmente nos bairros das áreas programáticas AP3 e AP5, mais pobres e com maiores taxas de sífilis gestacional e congênita.

**CONCLUSÕES:**

Analisar o desabastecimento de PB e sua evolução temporal e distribuição espacial no município do Rio de Janeiro permitiu reconhecer que os habitantes do município são afetados de diferentes modos. Compreender esse processo ajuda a planejar ações para enfrentar crises de desabastecimento, minimizando possíveis impactos no controle da sífilis, além de reduzir a desigualdade no acesso ao tratamento.

## INTRODUÇÃO

Entre 2014 e 2017 o Brasil vivenciou o desabastecimento das penicilinas benzatina (PB), procaína e cristalina, principais antibióticos para tratamento de sífilis que integram o Componente Básico da Assistência Farmacêutica (CBAF) no Sistema Único de Saúde (SUS). O medicamento esteve em falta no varejo farmacêutico e os poucos estoques disponíveis nos serviços do SUS foram destinados prioritariamente ao tratamento de sífilis, especialmente casos de gestantes e de sífilis congênita (SC) ^1^ .

A sífilis, causada pelo *Treponema pallidum* , é uma infecção sexualmente transmissível (IST) de caráter sistêmico, curável e exclusiva do ser humano. Quando não tratada durante a gestação, resulta em considerável quantidade de mortes fetais e neonatais, com alta probabilidade de transmissão vertical, principalmente nas fases primária e secundária ^2 , 3^ .

No Brasil, a taxa de detecção da sífilis em gestantes vem aumentando: em 2010 havia 3,5 casos de sífilis gestacional (SG) e 2,4 casos de SC para cada 1.000 nascidos vivos; em 2016 as taxas de detecção de SG e incidência de SC foram, respectivamente, de 12,4 e 6,8 casos para cada 1.000 nascidos vivos ^4^ .

O relatório da Organização Pan-Americana da Saúde (Opas) de 2016 informa que o aumento da incidência de SC pode ser atribuído ao aprimoramento do sistema de vigilância epidemiológica, à ampliação da distribuição de testes rápidos para diagnóstico, à falta de penicilina e ao fato de que quase metade das unidades básicas de saúde não tratam os pacientes, mas os encaminham para outros níveis de atendimento, gerando perda do paciente durante o processo ^5^ .

Segundo estudo realizado por Nurse-Findlay et al. ^6^ , entre 2014 e 2016, 39 países relataram escassez de PB, dentre os quais cinco pertencentes à América Latina (Brasil, Jamaica, Panamá, Suriname e Trindade e Tobago). No Brasil, assim como em outros países, a escassez de PB resultou de uma combinação de fatores: redução de formuladores de dose final (FDF) no mercado e interrupção do certificado de qualidade do insumo farmacêutico ativo (IFA) ^6 , 7^ .

Apesar do diagnóstico acurado e do tratamento eficaz e de baixo custo, a SC ainda é um sério problema de saúde pública responsável por muitas complicações perinatais. Além de ser a segunda causa infecciosa de natimortos em todo o mundo, a SC é um fator evitável de morbimortalidade infantil, que ocorre predominantemente nos países de baixa e média renda ^8^ .

O desabastecimento de medicamentos é sabidamente um dos obstáculos ao tratamento oportuno e adequado de doenças evitáveis como a sífilis, de forma que conhecer a distribuição e as características desse fenômeno pode minimizar possíveis consequências à saúde da população. Sendo assim, este artigo analisa o processo de desabastecimento da PB, caracterizando sua evolução temporal e distribuição espacial no município do Rio de Janeiro de 2013 a 2017.

## MÉTODOS

Trata-se de estudo ecológico misto (múltiplos grupos e abordagem temporal), cujas unidades de análise foram os bairros/regiões e os trimestres dos anos abordados. A população analisada foi o conjunto dos casos de SG notificados pelo Sistema de Informação de Agravos de Notificação (Sinan) do município do Rio de Janeiro, bem como a distribuição de PB para esses casos, por local de residência (bairro), entre 2013 e 2017. Participaram do estudo apenas unidades de saúde (US) da esfera municipal. Ou seja, foram excluídos registros de hospitais federais, estaduais, universitários, privados e psiquiátricos de outras esferas administrativas.

As unidades geográficas de estudo foram 160 bairros e suas respectivas áreas programáticas (AP) em saúde ( Figura 1 ) registrados no Sinan no período abordado. Foram utilizadas quatro fontes de dados: (i) bancos de dados de registros de SG e SC do Sinan do município do Rio de Janeiro; (ii) relatórios e planilhas de distribuição da PB disponibilizada pelo Núcleo de Assistência Farmacêutica (NAF) da Secretaria Municipal de Saúde (SMS-Rio); (iii) dados populacionais dos setores censitários e bairros do Censo Demográfico de 2010 do Instituto Brasileiro de Geografia e Estatística (IBGE); e (iv) dados de registros de nascidos vivos do Sistema de Informação de Nascidos Vivos (Sinasc) do município do Rio de Janeiro.

**Figura 1:** Mapa da divisão do município do Rio de Janeiro por Áreas Programáticas da Saúde.

Nota: Códigos dos bairros: 1 - Saúde; 2 - Gamboa; 3 - Santo Cristo; 4 - Caju; 5 - Centro; 6 - Catumbi; 7 - Rio Comprido; 8 - Cidade Nova; 9 - Estácio; 10 - Imperial de São Cristóvão; 11 - Mangueira; 12 - Benfica; 13 - Paquetá; 14 - Santa Teresa; 15 - Flamengo; 16 - Glória; 17 - Laranjeiras; 18 - Catete; 19 - Cosme Velho; 20 - Botafogo; 21 - Humaitá; 22 - Urca; 23 - Leme; 24 - Copacabana; 25 - Ipanema; 26 - Leblon; 27 - Lagoa; 28 - Jardim Botânico; 29 - Gávea; 30 - Vidigal; 31 - São Conrado; 32 - Praça da Bandeira; 33 - Tijuca; 34 - Alto da Boa Vista; 35 - Maracanã; 36 - Vila Isabel; 37 - Andaraí; 38 - Grajaú; 39 - Manguinhos; 40 - Bonsucesso; 41 - Ramos; 42 - Olaria; 43 - Penha; 44 - Penha Circular; 45 - Braz de Pina; 46 - Cordovil; 47 - Parada de Lucas; 48 - Vigário Geral; 49 - Jardim América; 50 - Higienópolis; 51 - Jacaré; 52 - Maria da Graça; 53 - Del Castilho; 54 - Inhaúma; 55 - Engenho da Rainha; 56 - Tomás Coelho; 57 - São Francisco Xavier; 58 - Rocha; 59 - Riachuelo; 60 - Sampaio; 61 - Engenho Novo; 62 - Lins de Vasconcelos; 63 - Meier; 64 - Todos os Santos; 65 - Cachambi; 66 - Engenho de Dentro; 67 - Água Santa; 68 - Encantado; 69 - Piedade; 70 - Abolição; 71 - Pilares; 72 - Vila Kosmos; 73 - Vicente de Carvalho; 74 - Vila da Penha; 75 - Vista Alegre; 76 - Irajá; 77 - Colégio; 78 - Campinho; 79 - Quintino Bocaiuva; 80 - Cavalcanti; 81 - Engenheiro Leal; 82 - Cascadura; 83 - Madureira; 84 - Vaz Lobo; 85 - Turiaçu; 86 - Rocha Miranda; 87 - Honório Gurgel; 88 - Oswaldo Cruz; 89 - Bento Ribeiro; 90 - Marechal Hermes; 91 - Ribeira; 92 - Zumbi; 93 - Cacuia; 94 - Pitangueiras; 95 - Praia da Bandeira; 96 - Cocotá; 97 - Bancários; 98 - Freguesia (Ilha); 99 - Jardim Guanabara; 100 - Jardim Carioca; 101 - Tauá; 102 - Moneró; 103 - Portuguesa; 104 - Galeão; 105 - Cidade Universitária; 106 - Guadalupe; 107 - Anchieta; 108 - Parque Anchieta; 109 - Ricardo de Albuquerque; 110 - Coelho Neto; 111 - Acari; 112 - Barros Filho; 113 - Costa Barros; 114 - Pavuna; 115 - Jacarepaguá; 116 - Anil; 117 - Gardênia Azul; 118 - Cidade de Deus; 119 - Curicica; 120 - Freguesia (Jacarepaguá); 121 - Pechincha; 122 - Taquara; 123 - Tanque; 124 - Praça Seca; 125 - Vila Valqueire; 126 - Joá; 127 - Itanhangá; 128 - Barra da Tijuca; 129 - Camorim; 130 - Vargem Pequena; 131 - Vargem Grande; 132 - Recreio dos Bandeirantes; 133 - Grumari; 134 - Deodoro; 135 - Vila Militar; 136 - Campo dos Afonsos; 137 - Jardim Sulacap; 138 - Magalhães Bastos; 139 - Realengo; 140 - Padre Miguel; 141 - Bangu; 142 - Senador Camará; 143 - Santíssimo; 144 - Campo Grande; 145 - Senador Vasconcelos; 146 - Inhoaíba; 147 - Cosmos; 148 - Paciência; 149 - Santa Cruz; 150 - Sepetiba; 151 - Guaratiba; 152 - Barra de Guaratiba; 153 - Pedra de Guaratiba; 154 - Rocinha; 155 - Jacarezinho; 156 - Complexo do Alemão; 157 - Maré; 158 - Vasco da Gama; 159 - Parque Columbia; 160 - Gericinó; 161 - Lapa; 162 - Vila Kennedy.

A medida de abastecimento de PB para uso na SG considerou a razão entre duas quantidades: (a) o número de frascos de PB dispensados para as unidades de saúde de cada bairro e (b) o número de frascos de PB necessários para tratar gestantes com sífilis e seus respectivos parceiros em cada bairro.

Os dados de distribuição de PB para as US de cada bairro do município do Rio de Janeiro foram extraídos de planilhas eletrônicas cedidas pela SMS-Rio, nas quais havia três tipos de informação: (i) número de frascos de PB dispensados para as US de cada bairro; (ii) número de frascos de PB dispensados para as Coordenadorias Gerais de Atenção Primária da Área Programática (CAP) e seus respectivos bairros de abrangência; (iii) número de frascos de PB dispensados especificamente para as US de cada bairro e para a CAP da mesma área de abrangência.

Para obter o número de frascos dispensados às US de cada bairro foram feitas as seguintes estimativas, de acordo com o tipo de dado: (i) para os dados do tipo 1 somou-se a quantidade de frascos dispensados para cada US que pertencia ao bairro; (ii) para o tipo 2, primeiro estimou-se, com base no número de notificações de SG, a quantidade de frascos distribuídos a cada US para, em seguida, somar a quantidade de frascos das US de cada bairro; (iii) quanto aos dados do tipo 3, foram combinados os dois métodos anteriores.

Como nem toda a PB que chegou às US destinou-se ao tratamento da SG, assume-se que 80% serviu ao tratamento de gestantes com sífilis e de seus parceiros. Essa premissa se justifica por dois motivos: (i) a utilização da PB para tratar outras infecções também prevalentes nas US, como a febre reumática, infecções do trato respiratório superior, infecções de tecidos moles (erisipela, impetigo) e pneumonias por *Streptococcus pneumoniae* sensíveis à penicilina; (ii) o uso de PB para conter o crescimento de casos de SC, visto que a SMS-Rio priorizou o tratamento das gestantes com sífilis com PB.

A fim de determinar a quantidade de frascos de PB necessários para tratar as gestantes e seus respectivos parceiros, foi considerada a dosagem de cada tratamento individual. Na falta de dados sobre a classificação clínica (estadiamento) da doença, considerou-se o esquema terapêutico com três doses de PB, cada qual utilizando dois frascos (1.200.000 UI), ou seja, seis frascos (7.200.000 UI) para o tratamento da gestante.

O número de frascos de PB necessários para tratar as gestantes notificadas foi calculado a partir do total de casos de SG notificados nas US de cada bairro, multiplicado por 6 e acrescido pela totalidade dos parceiros tratados, nos quais utilizou-se três doses de PB. Uma vez estimado o número de frascos dispensados e necessários, calculou-se a medida de abastecimento utilizando-se seguinte fórmula:

$$Ab_{i}=\frac{X_{i}}{Y_{i}}$$

*Ab*_i_ – Indicador de abastecimento do bairro *i* ;

*X*_i_ – Número de frascos de PB dispensados para o tratamento da SG e dos parceiros no bairro *i;*

*Y*_i_ *–* Número de frascos de PB necessários para tratar gestantes e parceiros no bairro *i.*

O indicador de abastecimento da PB foi calculado por trimestre, pois observou-se que a distribuição não era regular ao longo do ano. Com isso, considerou-se o trimestre a unidade de tempo, pois representava de maneira mais homogênea a distribuição de PB.

Como o indicador de abastecimento é uma razão cujos valores variam de 0 a ∞, para analisar o nível de abastecimento foram considerados os valores de *Ab* , bem como quatro importantes categorias: (i) grave, quando *Ab* teve valor entre 0 e 0,49; (ii) substancial, se *Ab* variou de 0,50 a 0,74; (iii) não substancial, se *Ab* variou entre 0,75 e 0,99 e (iv) sem desabastecimento, quando *Ab* foi maior ou igual a 1.

Para caracterizar os bairros, agruparam-se os serviços de saúde em quatro classes: (i) serviços de atenção primária (clínicas da família, centros municipais de saúde e equipes de saúde da família); (ii) serviços de atenção secundária (policlínicas e centro de atenção psicossocial); (iii) rede de urgência e emergência (unidades de pronto atendimento e coordenação de emergência regional); e (iv) hospitais.

Foram utilizadas informações sociodemográficas da população dos bairros, tais quais: razão homem-mulher, proporção da população vivendo em aglomerados subnormais, taxa de homicídio e Índice de Desenvolvimento Social (IDS). Em relação às características de saúde da população, utilizaram-se as taxas de nascidos vivos, de incidência de SC, de detecção de SG e a proporção de gestantes com teste não treponêmico realizado.

A evolução temporal do nível de abastecimento de PB no município foi analisada por meio de gráficos de tendência feitos no STATA 12.0. Para descrever a distribuição por trimestre/ano do desabastecimento de PB nos bairros do Rio de Janeiro, foram produzidos mapas temáticos no QGIS 2.18.14.

Por fim, os bairros foram analisados segundo os tipos de serviço de saúde (total), as condições sociodemográficas e as condições de saúde (média, mínima e máxima). Para caracterizar suas áreas, os bairros foram agregados segundo APs definidas pela SMS-Rio. Este projeto de pesquisa foi submetido e aprovado pelo Comitê de Ética em Pesquisa (CEP) da SMS-Rio (CAAE: 06019018.3.3001.5279).

## RESULTADOS

Entre 2013 e 2017 o município do Rio de Janeiro sofreu considerável desabastecimento de PB. Os anos de 2014 e 2015 tiveram os mais baixos níveis de abastecimento, ainda que em 2017 também se tenha observado níveis baixos, em comparação com os outros trimestres do período estudado ( Figura 2 ).

**Figura 2:** Nível de abastecimento de penicilina benzatina no município do Rio de Janeiro segundo trimestres-ano, de 2013 a 2017.

Nota: A linha tracejada indica razão de abastecimento igual a 1.

Se no início do período, especialmente em 2013, o nível de abastecimento de PB nos bairros cariocas é elevado, nos anos seguintes ocorre um decréscimo nos bairros da maioria das AP. O desabastecimento foi menor em 2013 e 2016, e mais frequente em 2014, 2015 e 2017, principalmente nos bairros das áreas programáticas AP3 e AP5 ( Figura 3 ).

**Figura 3:** Nível de abastecimento de penicilina benzatina por áreas programáticas e no município do Rio de Janeiro segundo trimestres-ano, de 2013 a 2017.

Nota: A linha tracejada indica razão de abastecimento igual a 1.

Os bairros das áreas AP2.1 e AP2.2 tiveram, em quase todos os trimestres do período estudado, níveis de abastecimento acima da média municipal, enquanto as demais áreas apresentaram níveis inferiores à média da cidade em 2014 a 2017. Os níveis de abastecimento foram mais baixos em 2014 e 2015, apesar de intenso desabastecimento em 2015 em bairros das AP3.1, AP3.2 e AP3.3 e, em 2017, nas AP5.1, AP5.2 e AP5.3 ( Figura 3 ).

A distribuição espacial do nível de abastecimento de PB nos bairros do Rio de Janeiro foi heterogênea em todos os anos estudados, mas há menor proporção de bairros com desabastecimento em 2013 e maior proporção em 2014. Em 2013, os bairros que apresentaram desabastecimento localizavam-se principalmente nas AP3.1, AP3.2 e AP3.3 ( Figura 4 ). Em 2014, especialmente no segundo trimestre, houve desabastecimento quase universal, apesar da maior concentração, nos demais trimestres, em bairros das AP3 e AP5, e de os bairros das AP2.1 e AP2.2 terem os menores níveis de desabastecimento.

**Figura 4:** Distribuição espacial do abastecimento de PB por bairros do município do Rio de Janeiro segundo trimestres-ano, de 2013 a 2017.

O desabastecimento observado em 2014 parece se estender até o segundo trimestre de 2015, diminuindo em 2016, principalmente nos dois trimestres do meio do ano. Mas, à semelhança dos demais anos, há aglomerados de bairros com desabastecimento nas AP3.1 e AP3.2. Em 2017, esta zona se amplia e intensifica, atingindo principalmente os bairros das AP5.2 e AP5.3. Em resumo, a distribuição do fenômeno em todos os períodos caracteriza-se principalmente pelas diferenças entre as zonas sul (AP2), com pouco ou nenhum desabastecimento, norte (AP3), com elevado desabastecimento, e oeste (AP5), com bairros fortemente desabastecidos, principalmente no fim do período estudado ( Figura 4 ).

Em relação às características das regiões do município do Rio de Janeiro, há diferenças na quantidade de US, particularmente quanto às unidades de atenção primária, que se concentram mais nas APs das zonas norte (AP3) e oeste (AP5), ainda que a primeira tenha mais bairros e seja mais populosa e a segunda seja maior e menos habitada ( Tabela ).

**Tabela:** Características das regiões (áreas programáticas) segundo tipos de serviço de saúde, condições sociodemográficas e de saúde do município do Rio de Janeiro entre 2013 e 2017.

Variáveis	AP 1.0	AP 2.1	AP 2.2	AP 3.1	AP 3.2	AP 3.3	AP 4.0	AP 5.1	AP 5.2	AP 5.3
Bairros (N)	15	18	7	28	23	29	19	10	8	3
										
**Serviços de Saúde (N)**										
Serviços de atenção primária	16	11	9	26	20	26	12	20	31	23
Serviços de atenção secundária	1	0	1	3	1	0	2	1	0	1
Rede de urgência e emergência	1	2	0	3	1	2	2	3	0	4
Hospital	5	2	3	2	3	4	4	1	0	0
Características da população*										
Sociodemográficas										
Razão Homem-Mulher	0,92	0,81	0,81	0,89	0,87	0,87	0,91	1,18	0,95	0,93
	(0,82 - 1,06)	(0,74 - 0,97)	(0,77 - 0,91)	(0,83 - 0,98)	(0,78 - 1,31)	(0,79 - 1,06)	(0,84 - 0,98)	(0,88 - 3,81)	(0,90 - 1,02)	(0,92 - 0,93)
Proporção da população vivendo em aglomerados subnormais	33,3	17,8	15,8	28,5	15,8	22,9	21,8	13,8	12,3	13,2
	(0 - 79,4)	(0 - 100)	(0 - 43,5)	(0 - 84,3)	(0 - 85,8)	(0 - 92,1)	(0 - 68,2)	(1,8 - 36,0)	(0 - 27,6)	(3,0 - 22,8)
Taxa de homicídio	119,4	33,3	47,1	71,9	81,9	128,9	85,1	94	94,8	110,0
	(38,8 - 224,3)	(0 - 89,4)	(7,9 - 137,4)	(0 - 229,8)	(25,4 - 226,4)	(18,6 - 404,3)	(22,8 - 175,0)	(6,6 - 175,2)	(39,2 - 189,7)	(88,4 - 137,1)
Índice de desenvolvimento social	0,59	0,71	0,65	0,60	0,61	0,59	0,58	0,58	0,57	0,54
	(0,55 - 0,63)	(0,54 - 0,8)	(0,54 - 0,7)	(0,53 - 0,71)	(0,54 - 0,68)	(0,54 - 0,65)	(0,31 - 0,76)	(0,55 - 0,64)	(0,5 - 0,72)	(0,53 - 0,55)
Saúde										
Taxa de nascidos vivos	244,6	112,6	60,6	107,3	151,8	82,4	164,1	96,7	86,3	32,4
	(14,6 - 1.327,6)	(1,7 - 930,5)	(44,9 - 83,3)	(0,15 - 727,6)	(8,9 - 532,9)	(1,7 - 485,5)	(2,8 - 1.524,5)	(0,2 - 275,1)	(0,06 - 273,4)	(3,2 - 74,8)
Taxa de incidência de sífilis congênita	23	13	13,8	428,9	16,3	17,8	15,7	18,7	2609,6	21,3
	(2,1 - 35)	(1 - 33,4)	(4,9 - 35,2)	(0,27 - 1.1494)	(2,2 - 54,2)	(2,9 - 44,3)	(0,76 - 42,8)	(4,2 - 30,4)	(4,6 - 1.8158)	(14,2 - 25)
Taxa de detecção de sífilis gestacional	47,1	26,8	23,7	1.061,6	39,7	38,3	30,3	42,3	3.066,3	40,1
	(10,4 - 109,7)	(4 - 65,8)	(6,1 - 81,8)	(3 - 2.8735)	(6,8 - 169,8)	(4,5 - 89,7)	(2,2 - 70,3)	(4,2 - 76,5)	(10,2 - 24.211)	(29,6 - 47,2)
Proporção de gestantes com teste não treponêmico realizado	0,58	0,58	0,82	0,70	0,66	0,65	0,38	0,64	0,41	0,47
	(0 - 0,83)	(0 - 1)	(0,62 - 1)	(0 - 1)	(0,33 - 0,90)	(0 - 0,91)	(0 - 1)	(0 - 0,85)	(0 - 0,65)	(0,40 - 0,59)

* Valores de média (mínimo - máximo).

Os bairros da zona sul (AP2) têm melhor IDS, menos violência, menor proporção de domicílios em aglomerados subnormais e menor razão homem/mulher. As piores situações socioeconômicas estão nas zonas norte e oeste ( Tabela ).

Quando comparados às demais áreas do município, os bairros da zona sul se diferenciam significativamente quanto às taxas de nascidos-vivos e à proporção de gestantes que fizeram exame laboratorial para diagnosticar sífilis na gestação. Esse indicador reflete a qualidade da vigilância das US localizadas nos bairros cariocas ( Tabela ).

## DISCUSSÃO

O desabastecimento de PB no município do Rio de Janeiro entre 2013 e 2017 não foi homogêneo no espaço ou no tempo. Apresentou maior intensidade em 2014 nos bairros da zona norte, enquanto os bairros da zona sul e da região centro-sul (que faz limite com esta) praticamente não o sofreram. Em 2017, outro desabastecimento ocorreu, mas desta vez incorporando também a zona oeste.

Entre os principais fatores que podem ter contribuído para tal cenário, destaca-se que, principalmente em 2014, tanto no Brasil como no exterior, houve desabastecimento da PB devido à falta da matéria-prima específica para produzi-la ^1 , 3 , 5^ . Uma das causas da falta de penicilina no mundo é a dependência de um pequeno número de fabricantes globais do IFA. Nos últimos dez anos ao menos cinco empresas abandonaram o mercado global de penicilina em busca de remédios mais rentáveis ^5^ .

Atualmente apenas quatro empresas produzem o princípio ativo da penicilina, uma austríaca e três chinesas. Recentemente, essas empresas limitaram sua produção a 20% da capacidade, pois o fármaco tornou-se muito barato. O Brasil, apesar de ter uma indústria farmacêutica forte, parou de produzir IFA para diversos medicamentos no início dos anos 1990, inclusive para a penicilina. A retração interna na produção de IFA faz com que atualmente 90% das necessidades do setor sejam supridas por importações ^11^ .

Além disso, no final de 2015 a Agência Nacional de Vigilância Sanitária (Anvisa) retirou o certificado de boas práticas do fornecedor de IFA para a produção de penicilina. Apenas no fim de 2016 foi concedida uma renúncia, permitindo a dois laboratórios FDF comprar o IFA. No entanto, como os FDF necessitaram de tempo para demonstrar equivalência e receber autorização de comercialização com o novo fornecedor, ambos atrasaram significativamente a produção e reentrada no mercado ^6 , 12^ .

A irregularidade no fornecimento de IFA, principal matéria-prima, assim como o fornecimento de produtos de qualidade fora dos padrões aceitáveis, parecem ser os principais motivos para o desabastecimento de medicamentos, na medida em que tendem a interromper o processo produtivo ^13^ .

Sabe-se que o problema aumenta quando se reduz ou se limita os fabricantes a um único fornecedor. No caso do Brasil, que não produz a maioria dos fármacos em escala industrial, a dependência do mercado internacional coloca-o em uma situação especialmente frágil ^13^ .

Segundo relatório publicado pelo Ministério da Saúde em julho de 2015, em quase metade dos estados brasileiros faltava PB. Em 11 deles (41%), majoritariamente nas regiões Norte e Nordeste, o estoque se esgotara. As demais áreas, entre as quais o estado do Rio de Janeiro, também estavam desabastecidas ^14^ .

Esperava-se que a produção de PB fosse regularizada a partir de julho de 2014, o que não ocorreu, mantendo a escassez do medicamento em 2016 e 2017. O desabastecimento da penicilina G benzatina chegou a atingir 61% dos estados brasileiros e, no caso da penicilina cristalina, atingiu 100% dos estados em março de 2016 ^1^ .

De forma semelhante ao cenário nacional, nosso estudo revela que os níveis mais baixos de abastecimento de PB no município do Rio de Janeiro ocorreram em 2014 e 2015. Porém, deve-se ressaltar que este se deu de forma heterogênea entre as áreas de planejamento do município, sendo mais evidente nas áreas das AP 3 e 5, zona norte e oeste, respectivamente.

As áreas de planejamento 3.1, 3.2 e 3.3 abrangem juntas metade dos bairros do município do Rio de Janeiro, além de apresentarem em média maiores taxas de homicídio e de nascidos vivos em relação às outras áreas. Já as áreas de planejamento 5.1, 5.2 e 5.3 possuem juntas a maior extensão territorial e, em média, têm as maiores taxas de SC e SG, bem como os menores valores para o IDS.

Estudo realizado por Domingues et al. (2014) ^15^ entre 2011 e 2012 com 23.894 mulheres brasileiras apontou que a ocorrência de sífilis foi 3,2 vezes maior entre as gestantes que possuíam até sete anos de estudo, em relação àquelas que possuíam maior grau de instrução. Mesquita et al. (2012) ^16^ avaliaram o perfil epidemiológico dos casos de sífilis em gestantes na cidade de Sobral (CE) no período de 2006 a 2010, e também demonstraram que a doença foi mais prevalente nas gestantes que cursaram apenas da 5ª à 8ª série do ensino fundamental.

Há ainda evidências de que a incidência de SC é mais elevada em estratos populacionais de menor escolaridade, em grupos raciais desfavorecidos socioeconomicamente (negros), e com piores condições de vida ^17 , 18^ . Tais fatores reforçam a ocorrência de casos de sífilis em gestantes com baixa escolaridade e em áreas com piores condições socioeconômicas em situações de crise, como o desabastecimento de PB, como se observou nesta pesquisa.

Outro fator relevante sobre as condições socioeconômicas e a sífilis é sua alta taxa de transmissão vertical. O estudo realizado por Domingues et al. (2014) ^19^ constatou uma taxa de transmissão vertical de 34,3%, na qual a chance de uma gestante ter o desfecho de SC foi dezesseis vezes maior naquelas com menor nível de escolaridade. Apesar da transmissão vertical da sífilis ter intensidade distinta conforme a fase da doença materna, sabe-se que o tratamento adequado com penicilina é capaz de prevenir 97% dos casos de transmissão vertical ^20^ . A falta do medicamento impacta o controle da SC, considerando as dificuldades da cobertura de triagem da sífilis na gestação e a aderência ao tratamento pela gestante e pelo parceiro.

Em 2016, no Brasil, 58,1% das gestantes com sífilis receberam tratamento inadequado, 26,5% não receberam tratamento, 11,3% dos casos foram ignorados e apenas 4,1% receberam a terapêutica adequada ^3^ . Deve-se ressaltar que a maioria das gestantes não tratadas ou não tratadas adequadamente pode transmitir a infecção para seus conceptos, ocasionando possível morte fetal ou neonatal, além de prematuridade, baixo peso ao nascer ou infecção congênita ^21^ .

Em 2016 Taylor et al. ^20^ estimaram a quantidade de PB necessária para tratar gestantes com diagnóstico de sífilis, a fim de enfrentar a epidemia de SC nos países com alta carga da doença. Os resultados desse estudo sugerem que esses países necessitam de uma quantidade de PB duas vezes maior do a prevista anualmente, caso sejam adotadas as recomendações da Organização Mundial de Saúde para a eliminação da SC, atingindo pelo menos 95% da cobertura para triagem da sífilis.

As principais limitações deste estudo estão relacionadas à disponibilidade dos dados de distribuição da PB nas US, do registro da distribuição na rede municipal de saúde e no próprio método utilizado para estimar o nível de abastecimento de PB. O aumento da incidência de SG e SC no período estudado indica que a subnotificação, apesar de plausível, não impediu a detecção da piora da situação epidemiológica. Também é improvável que tenham ocorrido variações importantes da suposta subnotificação no tempo e no espaço.

## CONSIDERAÇÕES FINAIS

Analisar o desabastecimento da PB e sua evolução temporal e distribuição espacial no município do Rio de Janeiro permitiu reconhecer que os habitantes do município são afetados de diferentes modos. Compreender esse processo permite planejar ações para enfrentar crises de desabastecimento, minimizando possíveis impactos no controle da sífilis, além de reduzir a desigualdade no acesso ao tratamento.

É evidente a necessidade de continuar a buscar soluções de longo prazo que assegurem a oferta da penicilina no SUS, garantindo enfrentamento adequado da sífilis e evitando novas crises de desabastecimento. A penicilina, nas formulações procaína e cristalina, é imprescindível para tratar a SC e outras infecções, reduzindo a mortalidade neonatal. Mais estudos sobre o tema são necessários, visto que o impacto do desabastecimento da PB no aumento significativo de SC tem sido pouco explorado.
